# Knowledge, attitudes and practices survey on antimicrobial resistance and stewardship among pharmacy healthcare workers in 28 African countries

**DOI:** 10.1136/bmjgh-2025-019151

**Published:** 2025-10-23

**Authors:** Oluoma Agiri, Gilbert Osena, Felix Bahati, Delaney Dill, Erta Kalanxhi, Diane Ashiru-Oredope, Yewande Habibat Alimi

**Affiliations:** 1 One Health Trust, Washington DC, District of Columbia, USA; 2 Infectious Diseases, University of Gothenburg Institute of Biomedicine, Goteborg, Sweden; 3 Centre for Global Health Research (KEMRI-CGHR), Kisumu, Kenya; 4 Commonwealth Pharmacists Association, London, England, UK; 5 UK Health Security Agency, London, UK; 6 Africa Centres for Disease Control and Prevention, Addis Ababa, Ethiopia

**Keywords:** KAP survey, Public Health, Health Personnel

## Abstract

**Introduction:**

Antimicrobial resistance (AMR) is a pressing global health problem disproportionately affecting low- and middle-income countries. Inappropriate antimicrobial prescription and use exacerbate AMR. This study assesses the knowledge, attitudes and practices (KAP) toward AMR and antimicrobial stewardship (AMS) among pharmacy healthcare workers involved in antimicrobial dispensing across 28 African countries.

**Methods:**

An online survey was distributed to collect data on KAP from HCWs who dispense antimicrobials in African countries. Responses were scored, and a 70% cut-off mark was used to differentiate between good and poor KAP. Logistic regression analysis was used to identify factors associated with good or poor KAPs.

**Results:**

A total of 2567 responses (40%) were received, of which 908 were from pharmacy HCWs who dispensed antibiotics in 28 countries. Of the 908 eligible respondents, 71.3% had good knowledge of AMR and AMS, 59.9% displayed good attitudes towards the burden of AMR and appropriate prescription of antimicrobials and 41.6% displayed good practices related to AMS. Patient demands and influence from pharmaceutical companies were among the factors that influenced the dispensing of antibiotics. In multivariable logistic regression, licensed pharmacists were more likely to have good knowledge of AMR than pharmacy technicians (adjusted OR (aOR) 1.78; 95% CI 1.64 to 1.93). Male dispensers were less likely to have a positive attitude towards AMR than female dispensers (aOR 0.69, 95% CI 0.51 to 0.94). Moreover, dispensers affiliated with public health facilities demonstrated better AMR practices than those affiliated with private facilities. Overall, good AMR knowledge status was significantly associated with positive attitudes (χ²=97.1, p<0.001) and practices (χ²=6.5, p<0.05) of AMR.

**Conclusion:**

This study revealed limited understanding of AMR among dispensers without formal pharmaceutical training and a positive association between good knowledge and positive attitudes and practices. The findings underscore the importance of providing workplace educational materials on AMR and AMS to build capacity in healthcare institutions and promote proper antibiotic dispensing.

**WHAT IS ALREADY KNOWN ON THIS TOPIC**

**WHAT THIS STUDY ADDS**

- We identified poor knowledge and practices related to AMR among HCWs with fewer years of job experience, highlighting a gap in formal education on AMR within tertiary institutions in Africa.
- The observations suggest that patient demands and pharmaceutical industry recommendations significantly impact antibiotic dispensing, often overlooking local or national treatment guidelines.
- Female dispensers were found to exhibit a more positive attitude towards AMR compared with their male counterparts.

**HOW THIS STUDY MIGHT AFFECT RESEARCH, PRACTICE OR POLICY**

- It is critical to increase access to educational materials on AMR and antimicrobial stewardship (AMS) in graduate and postgraduate curricula and the workplace.
- It is essential to include pharmacists in context-specific AMS programmes, acknowledging their important role as the primary point of contact for access to healthcare.
- Mentorship programmes with pharmacies affiliated with public health institutions could improve antibiotic dispensing practices in private pharmacy settings.

## Introduction

 Antimicrobial resistance (AMR) is a pressing global health issue that disproportionately affects people in low- and middle-income countries (LMICs).^1^ AMR emerges as microorganisms develop mechanisms to resist antimicrobials (including antibiotics, antivirals and antifungals), eventually rendering these medications ineffective. Due to AMR, many infections are becoming hard to treat, resulting in higher mortality, disability and treatment costs.^2^ According to 2019 estimates, about 27 deaths per 100 000 were attributable to AMR in African countries—the highest rate in the world.^3^ For countries in the WHO African region, 1.05 million deaths were associated with bacterial AMR in 2019; however, cumulative AMR-attributable deaths are projected to reach 6.63 million between 2025 and 2050.^4 5^ Despite being a natural phenomenon, AMR is largely driven by the misuse and overuse of antimicrobials, often influenced by socioeconomic factors such as a lack of access to diagnostic tools, quality antimicrobials and poor health literacy.^6^ Additional factors include the lack of education and awareness about antimicrobial stewardship (AMS) among healthcare professionals, which involves the optimisation of antimicrobial use through appropriate prescribing and management practices; the absence of stewardship programmes in healthcare facilities; and incentivisation by pharmaceutical companies.^27^,^10^

Healthcare workers (HCWs) who dispense antimicrobials can play an essential role in controlling the development and impact of AMR. They can achieve this through AMS by educating consumers on responsible drug use, verifying the quality of antimicrobials to prevent the dispensing of substandard or counterfeit drugs and advising physicians on the appropriate prescribing of antimicrobials.^9 11^ These are all actions that help improve healthcare quality and mitigate AMR. However, in many countries in Africa, these opportunities are often underused,^9^ retailers are unregulated and antimicrobials are commonly sold without prescription.^12 13^ Therefore, assessing the pharmacists’ and retailers’ knowledge, attitudes and practices (KAP) toward AMR and AMS can inform policies on antimicrobial prescription and dispensing, guide programmes on effective stewardship and help define the roles of pharmacists and retailers in healthcare settings regarding AMS.^14^ Additionally, evidence suggests that better knowledge and positive attitudes towards AMR can lead to increased adherence to AMS principles, such as rational antimicrobial prescribing and use.^15 16^ Previous KAP studies on pharmacists conducted in African countries, including Libya, Zambia and Nigeria, report that many pharmacists have a moderate perception of AMR, low knowledge of AMS and high interest in stewardship, respectively.^17 18^ However, the studies also identify gaps in AMS training, evaluation and auditing of prescription and dispensing practices.^14 17 19^

This study assesses the KAP towards AMR and AMS among vital players in antimicrobial dispensing, including pharmacists and drug retailers, across 28 countries in Africa. The findings from this study aim to inform policy and interventions on AMR and AMS within the AU.

## Methods

### Survey development

In 2021, technical experts from the Africa Union (AU) Taskforce on AMR led by the Africa Centres for Disease Control and Prevention (Africa CDC and African-Union Interafrican Bureau for Animal Resources), the One Health Trust, UK Health Security Agency and the Commonwealth Pharmacists Association developed a survey tool (online supplemental table 1) to assess KAP toward AMR among HCWs dispensing antibiotics, including pharmacists, pharmacy technicians or support staff and other actors such as drug store owners or staff, and pharmacy managers. The survey tool employed in this study was specifically designed for healthcare professionals engaged in antimicrobial dispensing. It incorporated AMR knowledge queries from the WHO tool, the Multi-Country Public Awareness Survey,^20^ alongside questions tailored to the specific context of this study to ensure a more accurate representation of the participants’ understanding and practices on AMR-related issues.

The survey (translated into two additional AU working languages, French and Arabic) was pilot-tested on a focus group of four individuals from the target stakeholder group to determine the content validity of the questionnaire in assessing KAP among the antimicrobial dispensers. This was a cross-sectional survey, in which participants answered structured, closed-ended questions regarding their demographics, KAP and education and training (online supplemental table 1). The survey required approximately 25 min to complete.

### Survey population and data collection

Using a convenience sampling technique, Africa CDC distributed the survey link via email from 30 June to 5 July 2022, to 6412 participants in the AU database of registered HCWs (prescribers and dispensers) in AU member states. Emails in English and French encouraged HCWs to share the survey link with their colleagues, applying snowball sampling and leveraging their networks. The survey was created and administered using the Kobo Toolbox Software. The data collection period ran until 1 August 2022.

### Scoring

The survey comprised 28 knowledge, 9 attitudes and 9 practices questions. The cut-off score indicating good KAP was 70%.^21^,^23^ The maximum knowledge score was 28, with a score of 19 or higher (≥ 70%) considered to reflect good knowledge. Attitude questions were based on a Likert scale, where each answer was awarded between 1 and 5 points, and the maximum score for all questions consisted of 41 points. Scores above 70% (28/41) were considered to reflect positive attitudes. Practices questions were awarded a total of 36 points, with scores above 70% (25/36) reflecting good practices. The decision to use the ≥70% cut-off value stems from its application in numerous previous KAP studies, where it serves as a widely accepted criterion for evaluating good performance in educational and survey research.^24^

### Statistical analysis

Summary statistics describing the population characteristics and the frequency of responses to the KAP survey questions according to AU member states and regions were performed using averages. Spearman’s correlation test was performed to check for collinearity among the demographic (independent) variables. We chose a coefficient of 0.6 as a threshold for indicating a moderate relationship between the variables (online supplemental tables 6,7). The associations between demographic variables and KAP components were separately analysed in a multivariable logistic regression model. The models were evaluated for goodness of fit using the Hosmer and Lemeshow test.^25^ To account for potential intracluster correlation within the different regions, which could lead to underestimation of standard errors in the multivariable logistic regression, we estimated cluster-robust SEs using the cluster-robust variance–covariance estimator. Associations between KAP scores were analysed using the χ² test. Missing data were assumed to be missing at random and were addressed using the transcan multiple imputation function in the ‘Hmisc’ package of R software (V.4.3.0). Missing data occurred across different variables and was less than 1% of the entire data set. The analysis was performed using R software (V.4.3.0) at a 95% significance level.

### Patient and public involvement

Before dissemination, the survey was pilot-tested on a focus group of four HCWs dispensing antibiotics. The focus group provided feedback on how the questions were framed, their relevance and additional suggestions to improve the questionnaire. Following the piloting of the questionnaire, additional questions were added to inquire about pharmacy HCWs’ familiarity with popular AMR terminology and how they access information about antimicrobials. Furthermore, questions were added to inquire about whether the HCWs had received training on substandard or falsified medicines, the frequency with which they encountered suspected substandard or falsified medicines and whether they knew how to report them. Professionals who provided feedback during the piloting phase were excluded from the final survey. The survey was publicly shared with all HCWs registered in the AU database, who were encouraged to share it with their network. We aim to share the findings of this study through press releases and open-access publications.

## Results

### Sociodemographic characteristics of the respondents

Overall, we received 2567 responses to the survey, reflecting a response rate of 40%, 1659 of which were excluded from the analysis due to being from non-pharmacy HCWs. Being a cross-sectional survey, participation and completion were similar. Of the 908 individuals across 28 countries in Africa (online supplemental table 2), the majority were licensed pharmacists (46.6%) and pharmacy technicians (23.2%) (table 1). Pharmacy managers and store owners comprised 14.0% and 12.6% of respondents’ occupations, and only 1.0% (10) were other types of HCW or support staff. Most respondents worked in the private sector, in independent pharmacies (36.8%), chain private pharmacies (10.1%) or pharmacies associated with a private health facility (13.9%). Only 16.9% worked in pharmacies or dispensaries within public health facilities.

**Table 1:** Sociodemographic characteristics of the respondents

Variable	Number of respondents (%)
Gender (N=900)	
Male	545 (60.6)
Female	354 (39.3)
Other	1 (0.1)
Age (N=905**)**	
18–24	242 (26.7)
25–34	413 (45.6)
35–44	156 (17.2)
45–54	69 (7.6)
54 years or older	25 (2.8)
Job title (N=906**)**	
Licensed pharmacist	422 (46.6)
Pharmacy technician or technician manager	210 (23.2)
Pharmacy manager	127 (14.0)
Drug store owner or staff	114 (12.6)
Licensed pharmacist manager	23 (2.5)
Other HCW or support staff	10 (1.1)
Place of work (setting) (N=907**)**	
Independent private pharmacy	334 (36.8)
Pharmacy or dispensary within a public health facility	153 (16.9)
Pharmacy or dispensary within a private health facility	126 (13.9)
Chain private pharmacy, medical store or dispensary	92 (10.1)
Pharmacy or dispensary within a faith-based, or non-government facility	55 (6.1)
Multiple facilities	47 (5.2)
Other	43 (4.7)
Central medical store	30 (3.3)
University	15 (1.7)
Professional body	12 (1.3)
Work experience (years) (N=906**)**	
≤1	139 (15.3)
2–5	422 (46.6)
6–10	184 (20.3)
11–15	81 (8.9)
>16	80 (8.8)

HCW, healthcare worker.

The largest group of respondents consisted of individuals aged between 25 and 34 years (45.6%), followed by those aged 18 and 24 years (26.7%). Overall, most respondents had between 2 and 10 years of work experience, with only 80 (8.8 %) having more than 15 years of experience (table 1). Among the independent variables, the correlation between ‘Age’ and ‘Years of Experience’ variables exceeded the 0.6 threshold set for non-collinearity (online supplemental table 6); therefore, only ‘Years of Experience’ was retained as a variable for the regression analysis.

### Knowledge of antimicrobial resistance and stewardship

We observed that 71% (647) of the respondents demonstrated good knowledge of AMR and AMS. Although nearly all participants correctly identified that antibiotics are used to treat bacterial infections (96.7%, 878), 26.3% believed that antibiotics could also treat viral diseases (online supplemental table 3). Further, 80.8% (734) were aware that even healthy individuals can transmit antibiotic-resistant bacteria, and 91% (827) recognised that such bacteria are difficult or impossible to treat (online supplemental table 3). A considerable proportion of dispensers were knowledgeable about AMR control strategies, including the use of vaccines (60.2%, 547) and the importance of good sanitation and hygiene (79.5%, 722) (online supplemental table 3).

### Attitudes on the burden of AMR and appropriate prescription of antimicrobials

**Figure 1:**
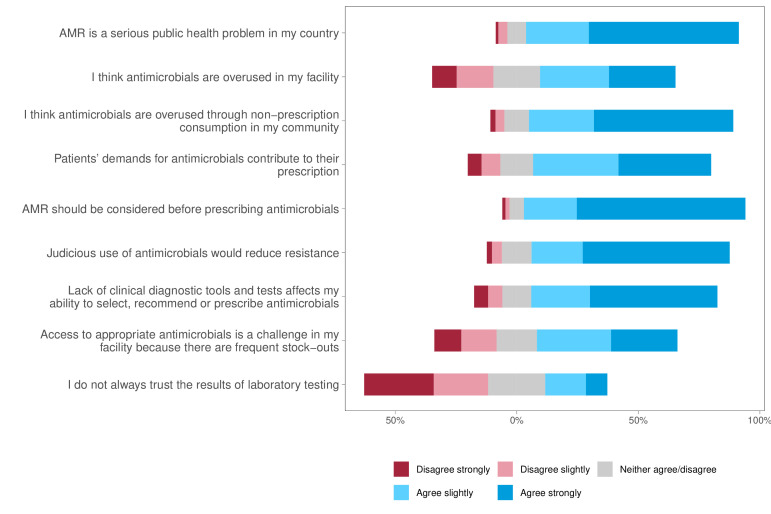
Distribution of participants’ attitudes on the burden of AMR and appropriate prescription of antimicrobials. AMR, antimicrobial resistance.

Approximately two-thirds (59.9%, 544) of the respondents displayed good attitudes towards AMR and appropriate prescription of antimicrobials, and most respondents (86.7 %) perceived AMR as a serious public health problem in their country (figure 1). Furthermore, 68.5% ‘strongly’ believed that AMR should be considered before antimicrobials are prescribed, and 53.4% were ‘very confident’ that they used antimicrobials optimally. However, 37.7% strongly agreed, and 34.7% slightly agreed that the patients’ demands influenced their choice of antimicrobial prescription (figure 1, online supplemental table 4). When asked about access to clinical diagnostic tools and tests, over half of the responses (52%) agreed that the lack of such tools affected their ability to appropriately select and dispense antimicrobials to patients. Nearly half of the respondents (46%) agreed that some antibiotics they sold were of poor quality and ineffective (figure 1, online supplemental table 4).

### Practices on antimicrobial stewardship

**Figure 2:**
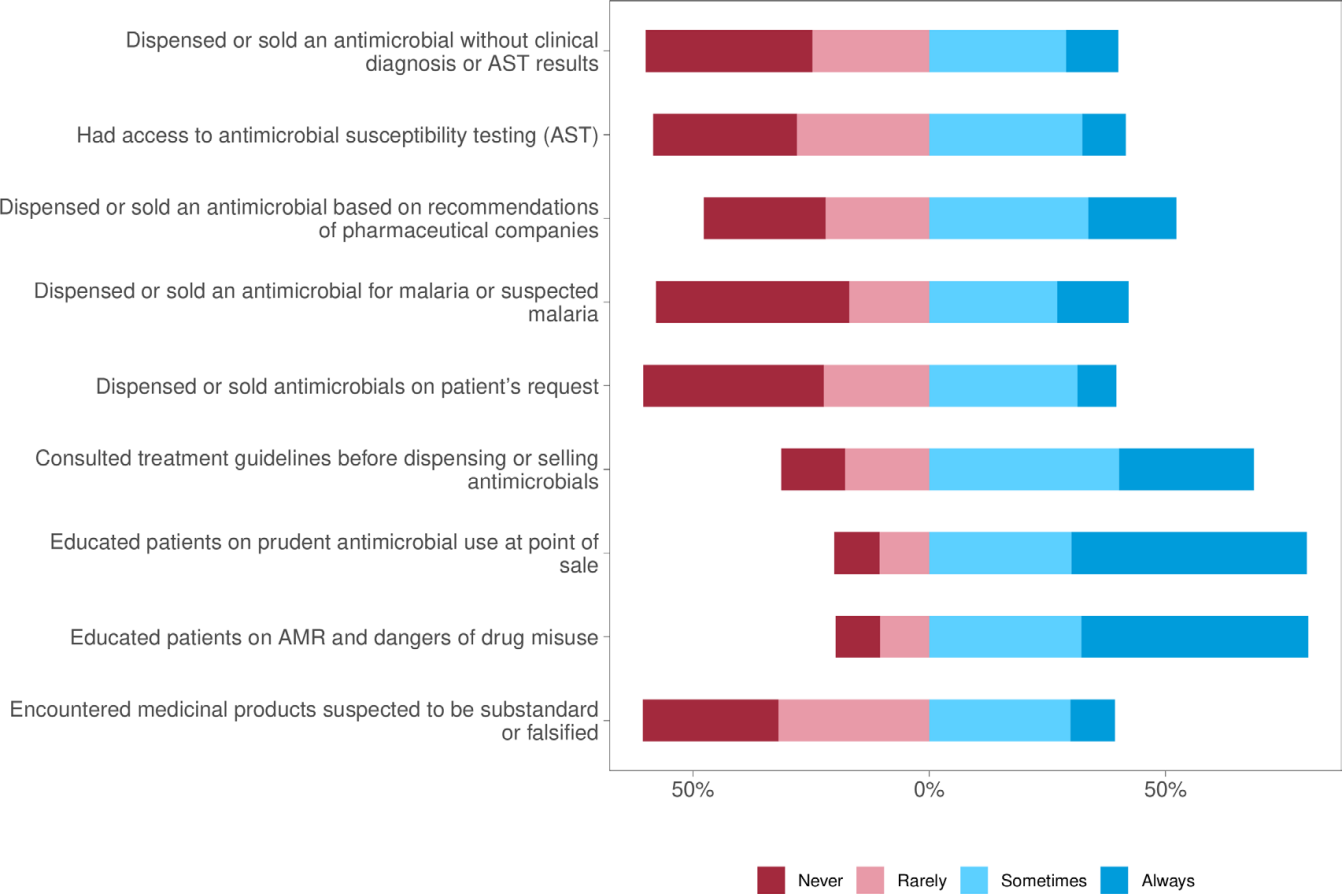
Distribution of participants’ practices on antimicrobial prescription and stewardship. AMR, antimicrobial resistance.

Overall, less than half of the respondents (41.6%, 378) displayed good practices related to AMS. Nearly half of the respondents (442, 48.7%) reported that the facility where they worked had access to an AMS committee, 479 (52.8%) had facility or local treatment guidelines and 445 (49%) had national treatment guidelines (online supplemental table 5). When asked whether they participated in AMS activities in their facilities, 60% (523) of respondents indicated participation, 20% (187) stated that they did not participate and the remaining 20% (180) reported that stewardship activities were lacking in their facilities (online supplemental table 5). While 43 (4.7%) reported limited knowledge of AMS, 27 (3%) believed that AMS was not their concern but instead in the healthcare provider’s domain and 10 (1%) stated that they thought AMS activities would not make much of a difference (online supplemental table 5). When questioned on their antibiotic dispensing practices, half of the respondents reported dispensing antibiotics based on recommendations of pharmaceutical companies (33.7%, ‘sometimes’; 18.6%, ‘always’), and 40% dispensed antibiotics based on patient requests, with 31.4% responding ‘sometimes’ and 8.2% responding ‘always” (figure 2). The majority (80%) reported they educated patients on prudent antibiotic use, with 49.8% responding that they always advised patients (figure 2).

### Association between sociodemographic variables and knowledge, attitudes and practices

In multivariable logistic regression, after adjusting for all the other independent variables, years of experience, job title and job settings were significantly associated with the level of AMR knowledge (table 2). Compared with pharmacy technicians, licensed pharmacists were more likely to have good knowledge of AMR (adjusted OR (aOR) 1.78; 95% CI 1.64 to 1.93). Dispensers attached to multiple facilities were five times (aOR 5.29, 95% CI 3.09 to 9.08) more likely to have good knowledge of AMR compared with those in independent private pharmacies. Respondents with more than ten years of experience were almost three times more likely to have good knowledge than those with less than 1 year of experience (aOR 2.98, 95% CI 1.70 to 5.24). The fitted logistic model had a goodness of fit metric of χ² = 13, p=0.112 (online supplemental figure 1).

**Table 2:** Multivariable logistic regression

Variable (N)	Knowledge		Attitude		Practice	
	Good (%)	aOR (95% CI)	Positive (%)	aOR (95% CI)	Good (%)	aOR (95% CI)
Gender						
Female (359)	71.9	Ref.	64.1	Ref.	45.4	Ref.
Male (549)	70.9	0.89 (0.78 to 1.01)	57.2	0.69 (0.51 to 0.94)*	39.2	0.81 (0.72 to 0.91)***
Years of experience						
≤1 (139)	64.0	Ref.	60.4	Ref.	37.4	Ref.
2–5 (423)	67.4	1.18 (0.89 to 1.57)	53.0	0.80 (0.61 to 1.06)	39.0	1.09 (0.87 to 1.37)
6–10 (184)	74.5	1.62 (1.07 to 2.44)*	57.1	0.87 (0.4 to 1.88)	39.1	1.03 (0.79 to 1.33)
11–15 (82)	84.1	2.98 (1.7 to 5.24)***	82.9	3.05 (1.63 to 5.71)***	52.4	1.66 (1.07 to 2.59)*
>16 (80)	83.8	2.79 (1.56 to 4.96)***	78.8	2.10 (0.81 to 5.48)	57.5	1.93 (0.85 to 4.39)
Job title						
Pharmacy technician (213)	62.4	Ref.	56.8	Ref.	44.1	Ref.
Drug store owner or staff (114)	67.5	1.25 (0.82 to 1.92)	48.2	0.70 (0.42 to 1.19)	34.2	0.67 (0.51 to 0.9)**
Licensed pharmacist (426)	77.2	1.78 (1.64 to 1.93)***	64.8	1.16 (0.77 to 1.73)	39.7	0.74 (0.47 to 1.18)
Pharmacy manager (155)	69.7	1.09 (0.88 to 1.36)	59.4	0.81 (0.48 to 1.36)	49	1.01 (0.6 to 1.71)
Job setting						
Independent private pharmacy (334)	68.6	Ref.	53.3	Ref.	34.4	Ref.
Chain private pharmacy, medical store or dispensary (93)	64.5	0.81 (0.61 to 1.07)	38.7	0.53 (0.41 to 0.69)***	38.7	1.16 (0.63 to 2.14)
Multiple facilities (47)	93.6	5.29 (3.09 to 9.08)***	85.1	4.45 (2.82 to 7.04)***	40.4	1.25 (0.63 to 2.47)
Pharmacy or dispensary within a public health facility (153)	71.9	0.94 (0.67 to 1.32)	73.9	1.99 (1.01 to 3.94)*	60.1	2.58 (1.62 to 4.12)***
Pharmacy or dispensary within a private/FBO/NGO health facility (181)	72.4	1.16 (0.66 to 2.05)	59.1	1.21 (0.67 to 2.2)	42.0	1.34 (0.99 to 1.8)
University/professional body/central store (57)	73.7	1.12 (0.95 to 1.31)	73.7	2.13 (1.12 to 4.05)*	31.6	0.81 (0.49 to 1.34)
Other (43)	72.1	0.83 (0.65 to 1.05)	65.1	1.06 (0.82 to 1.35)	51.2	1.55 (0.96 to 2.48)

Statistical significance: *p<0.05, **p<0.01, ***p<0.001.

aOR, adjusted OR; FBO, faith-based organisation; N, number of responders; NGO, non-governmental organisation; Ref, reference category.

Male dispensers were less likely to have a positive attitude towards AMR than their female counterparts (aOR 0.69, 95% CI 0.51 to 0.94). Dispensers working in pharmacies or dispensaries within a public health facility were two times (aOR 1.99, 95% CI 1.01 to 3.94) more likely to have a positive attitude towards AMR than those in independent private pharmacies. Additionally, dispensers affiliated with multiple facilities (aOR 4.45, 95% CI 2.82 to 7.04), as well as those linked to universities, central medical stores or professional bodies (aOR 2.1, 95% CI 1.12 to 4.05), were more likely to have a positive attitude compared with their counterparts in independent private pharmacies. However, dispensers in chain private pharmacies, medical stores or dispensaries were less likely to have a positive attitude than independent private pharmacies. The fitted logistic model had a goodness of fit metric of χ² = 6.26, p=0.619 (online supplemental figure 2).

Dispensers with more than 10 years of experience were at least 1.5 times more likely to follow good AMR practices than those with less than 1 year of experience. Furthermore, dispensers working in pharmacies or dispensaries within public health facilities were 2.5 times more likely to have a positive attitude toward AMR (aOR 2.58, 95% CI 1.62 to 4.12) than those in independent private pharmacies. The fitted logistic model had a goodness of fit metric of χ² = 6.73, p=0.566 (online supplemental figure 3).

Regarding regional distribution (online supplemental tables 8,9), average knowledge scores were highest in southern (mean percentage 80.3, 95% CI 77.3 to 83.3) and eastern sub-Saharan Africa (mean percentage 76.6, 95% CI 75.5 to 77.6) (online supplemental figure 4). Knowledge and attitude scores varied significantly across the different regions (p<0.001) (online supplemental figure 5). Practice scores also differed across the regions (p=0.01), with southern Africa recording the highest practice scores (mean percentage 72.5, 95% CI 70.0 to 75.1) (online supplemental figure 6).

Lastly, we examined the relationship between AMR knowledge and dispensers’ attitudes and practices related to AMR (online supplemental table 10). We found that a good level of AMR knowledge was significantly associated with both a positive attitude toward AMR (χ² = 97.1, p<0.001) and adherence to good AMR practices (χ² = 6.5, p<0.05). Separately, a positive attitude was strongly associated with good AMR practices (χ² = 68, p<0.001).

## Discussion

To our knowledge, this is the largest KAP study on AMR and AMS among pharmacy HCWs in Africa, including data from 908 HCWs across 28 African countries. Our findings reveal significant knowledge gaps associated with both the level of HCWs’ experience and the affiliation of pharmacies with healthcare facilities. Many respondents who lacked formal training in medication dispensing, such as drug store owners and unlicensed technicians, exhibited poor KAP related to AMR, highlighting the need for licensed personnel with adequate stewardship knowledge and skill to dispense antimicrobials. These findings align with previous studies^26 27^ and underscore the need to regulate unqualified personnel’s dispensing and prescription activities in healthcare provision, particularly in low-resource settings. Furthermore, knowledge of AMR and AMS increased with years of experience, suggesting possible gaps in the educational curricula of health professions while highlighting the importance of practices and continuing education. A similar observation was reported by Feng and colleagues among community pharmacists in North Eastern China.^28^

HCWs’ attitudes towards AMR and AMS are critical in AMR control since positive attitudes towards AMR and stewardship can lead to good dispensing practices and stronger motivation for self-development in stewardship.^29^ In this study, most respondents (86.5%) agreed that AMR was a serious public health problem in their countries, hinting at good awareness of the scope of the problem. Similar trends have been reported in other African countries like South Africa and Kenya, where most respondents agree that AMR is a national and global health issue.^26 30^ Most respondents (75.9%) believed AMS could improve the quality of medical care; however, only 57.7% stated that AMS would control AMR, possibly due to a lack of an in-depth understanding of AMS. More than half of the respondents in this study (57.6%) affirmed that they practised AMS; however, additional questions into the dispensing practices of the respondents revealed that only close to one-third (28%) of the respondents ‘always’ consulted treatment guidelines on antimicrobial prescription before dispensing to patients.

Our findings suggest an important role for patients’ demands for antimicrobials and recommendations from pharmaceutical companies in dispensing practices. This is in agreement with another study in Indonesia, where a higher population of respondents indicated that patient demand was a significant factor influencing medication sales and the overuse of antibiotics.^31^ Similar findings have been identified in Kenya, Tanzania and other LMICs.^2632^,^34^ Many factors can drive the dispensing of antibiotics without prescriptions, including incentivisation from pharmaceutical companies, poor healthcare access and high out-of-pocket expenses related to medical consultation. These findings underscore the importance of public education on appropriate antibiotic use and the dangers of AMR as an underused intervention in low-resource settings. Despite the low knowledge and awareness of AMR and AMS, most HCWs advised patients on the correct use of antibiotics, highlighting the potential that pharmacists can have on AMS.

The global action plan on AMR emphasises the importance of AMS training in professional and academic programmes and continuing education.^35^ AMS is effectively delivered through standardised and systematic training and learning complemented by professional practices to ensure quality control. AMS activities, such as using treatment guidelines and following an approval and review process for restricted antibiotics, must be ideally introduced to HCWs through undergraduate and postgraduate curricula and then implemented in practice. In this study, only half of respondents reported receiving AMS training during formal education. This may also explain our findings that dispensers in pharmacies linked to healthcare facilities, especially those in the public domain, displayed better knowledge of AMR and AMS. Supporting the need for more training on AMR and AMS, virtually all respondents indicated that additional training would be beneficial. These findings are similar to those from studies in South Africa and Kenya,^30 36 37^ where less than 50% of respondents had undergone training and the majority highlighted the necessity for more training.

Our study has some limitations. First, the survey was deployed to a set number of contacts across the AU member states to increase the study’s geographical coverage without sample size consideration to ensure equal representation of populations across countries. To overcome some of this bias that could result from regional intracorrelations, we applied cluster-robust standard errors using regions as a clustering variable to allow valid inference from the regression models. The sociodemographic characteristics of our sample are similar to those of pharmacy HCWs across the continent, which supports some generalisation of our findings. However, our convenience sampling method reduces the representativeness of this study to the broader population of pharmacy HCWs, and therefore, generalisations of our findings should be interpreted with caution. This could be due to the systematic exclusion of certain groups, such as HCWs without access to the internet or an email address. Second, HCWs working in facilities without AMS programmes may have been less likely to respond, potentially resulting in non-response bias. This could have resulted in gaps in our findings, especially regarding practices related to AMS, such as whether HCWs consulted treatment guidelines before dispensing antimicrobials, and should guide the degree of representativeness applied in this regard. Finally, as the responses were received via an online survey, there may be a certain degree of reporting bias, where the respondents might under-report undesirable answers regarding their knowledge, practices and attitudes related to AMR and AMS. This was particularly evident in responses to follow-up questions that contradicted initial answers regarding KAPs. For instance, some respondents stated that they practised AMS but later contradicted this when asked more about how often they consulted treatment guidelines before dispensing.

A recommendation derived from this study includes designing interventions to increase access to and implementation of available educational material on AMR and AMS, such as the WHO Open Academy AMS—a competency-based approach, British Society for Antimicrobial Chemotherapy Massive Open Online Course and Fleming Fund e-learning modules on AMR in graduate and postgraduate curricula and the workplace. Additionally, targeted interventions can be aimed at younger professionals with less experience and HCWs working in independent private pharmacies. More broadly, the responses from the HCWs in this study reflect the need for capacity building in healthcare institutions to ensure that HCWs have the necessary tools to practise AMS. Furthermore, the findings underscore the importance of increasing public awareness of AMR and antibiotic use, enforcing regulations on antibiotic sales and expanding universal healthcare coverage to increase healthcare access and reduce the patient demand for antibiotic dispensing without prescriptions.

## Conclusion

The majority of pharmacy HCWs surveyed in this study demonstrated a good understanding of AMR and AMS and recognised AMR as a serious public health issue. However, significant gaps were found in the effective practice of AMS, particularly among those with fewer years of experience. Furthermore, patient demands and pharmaceutical companies’ incentives were reported to increase the pressure to dispense medication without an appropriate prescription. In light of these findings, it is recommended that AMS education be enhanced and expanded across the continent in graduate, postgraduate and healthcare provision settings. Additionally, partnering pharmacies with public health institutions through mentorship programmes could lead to improvements in antibiotic dispensing practices across private facilities.

## Supplementary material

10.1136/bmjgh-2025-019151 online supplemental table 1

## Data Availability

All data relevant to the study are included in the article or uploaded as supplementary information.
