# Supplementary material for: Knowledge, attitudes and practices survey on antimicrobial resistance and stewardship among pharmacy healthcare workers in 28 African countries
Source: BMJ Glob Health. 2025 Oct 23;10(10):e019151. doi: 10.1136/bmjgh-2025-019151 (PMC12551537; doi:10.1136/bmjgh-2025-019151)
Supplement: online supplemental table 1 [file bmjgh-10-10-s001.docx]

Supplementary Materials

Table of Contents

[Supplementary Table 1. Survey questions and options for responses. 2](#_Toc202185128)

[Supplementary Table 2. List of countries included in the survey (Regions based on the classification of global burden of disease) 13](#_Toc202185129)

[Supplementary Table 2.1 List of countries with fewer than five responders 13](#_Toc202185130)

[Supplementary Table 2.2. List of countries with more than five responders 13](#_Toc202185131)

[Supplementary Table 3: Knowledge questions and responses scored and analysed in logistic regression 14](#_Toc202185132)

[Supplementary Table 3.1: True/False questions (Q36-Q50) 14](#_Toc202185133)

[Supplementary Table 3.2 Multiple choice knowledge questions (Q23-Q35) 15](#_Toc202185134)

[Supplementary Table 4: Attitude questions and responses 15](#_Toc202185135)

[Supplementary Table 5: Practice questions and responses. 19](#_Toc202185136)

[Supplementary Table 6: Spearman’s correlation matrix 22](#_Toc202185137)

[Supplementary Table 7: Univariate logistic regression 23](#_Toc202185138)

[Supplementary Table 7.1 Univariate regression analysis of knowledge 23](#_Toc202185139)

[Supplementary Table 7.2 Univariate Regression Analysis of Attitude Scores 24](#_Toc202185140)

[Supplementary Table 7.3 Univariate regression analysis of Practice Scores 25](#_Toc202185141)

[Model metrics 26](#_Toc202185142)

[Figure S1: Knowledge. Residuals plot (a) and calibration plot (b) for the AMR knowledge multivariable logistic regression model. The error bars on the calibration plot represent the 95% confidence interval (CI). 26](#_Toc202185143)

[Figure S2: Attitude. Residuals plot (a) and calibration plot (b) for the AMR attitude multivariable logistic regression model. The error bars on the calibration plot represent 95% CI. 27](#_Toc202185144)

[Figure S3: Practice. Residuals plot (a) and calibration plot (b) for the AMR practices multivariable logistic regression model. The error bars on the calibration plot represent 95% CI. 28](#_Toc202185145)

[Supplementary Table 8: Distribution of knowledge, attitude, and practice AMR scores across Sub-Saharan Africa (SSA) regions 29](#_Toc202185146)

[Supplementary Table 9: Pairwise Comparisons of knowledge, attitude, and practice mean scores across African regions: Tukey's Honestly Significant Difference (HSD) test 29](#_Toc202185147)

[Pairwise plots of Mean Differences in AMR Knowledge, attitude, and practice scores across African regions. 30](#_Toc202185148)

[(The reported mean difference is calculated by subtracting the mean of the second group from that of the first (e.g., for Southern vs. Western SSA, the mean for Western SSA is subtracted from the mean for Southern SSA) 30](#_Toc202185149)

[Figure S4: Knowledge 30](#_Toc202185150)

[Figure S5: Attitude 30](#_Toc202185151)

[Figure S6: Practice 31](#_Toc202185152)

[Supplementary Table 10: Association between distributors’ knowledge, attitude and practice 31](#_Toc202185153)

## Supplementary Table 1. Survey questions and options for responses.

| **Questions** | |
| --- | --- |
|  | |
| **Demographics** | |
| ***Variable*** | ***Options*** |
| Gender | Male |
|  | Female |
|  | Other |
|  | Did not answer |
|  |  |
| Age Category | 18-24 |
|  | 25-34 |
|  | 35-44 |
|  | 45-54 |
|  | 54 years or older |
|  | Did not answer |
|  |  |
| Job Title | Pharmacy technician or support staff |
|  | Pharmacist |
|  | Drug store owner or staff |
|  | Pharmacy manager |
|  | Other |
|  | Did not answer |
|  |  |
| Setting |  |
|  | Central Medical Store |
|  | Chain private pharmacy, medical store, or dispensary |
|  | Multiple Organizations |
|  | Pharmacy or dispensary within a public health facility |
|  | Independent private pharmacy |
|  | Pharmacy or dispensary within a faith-based, or non- government facility |
|  | Pharmacy or dispensary within a private health facility |
|  | Other |
|  | Did not answer |
|  | Professional Body |
|  | University |
|  |  |
| Years of Experience | 1 year or less |
|  | 2-5 years |
|  | 6-10 years |
|  | 11-15 years |
|  | More than 15 years |
|  | Did not answer |
|  |  |
| Role in dispensing antimicrobials | Stock management |
|  | Policy, regulations |
|  | Dispense or prescribe |
|  | Sell |
|  | Administer but do not prescribe |
|  | Support clinician-decision making |
|  | Other |
|  |  |
| Location of Education | In the same country where I work |
|  | Abroad (Africa) |
|  | Abroad (Europe) |
|  | Abroad (North America) |
|  | Abroad (Other) |
|  | Did not answer |
|  |  |
| **Knowledge** | |
|  | |
| **Ǫuestion** | **Options** |
| Does the country where you work allow you to prescribe medicines to patients? |  |
|  | I don't know |
|  | No |
|  | Yes |
| Which of the following terms or activities have you heard of before? Select all that you are familiar with | Antibiotic resistance |
| Which of the following terms or activities have you heard of before? Select all that you are familiar with | Antimicrobial resistance |
| Which of the following terms or activities have you heard of before? Select all that you are familiar with | Antimicrobial stewardship |
| Which of the following terms or activities have you heard of before? Select all that you are familiar with | Access, Watch, Reserve (AWaRe) |
| Which of the following terms or activities have you heard of before? Select all that you are familiar with | Antimicrobial susceptibility |
| Which of the following terms or activities have you heard of before? Select all that you are familiar with | Judicious antimicrobial use |
| Which of the following terms or activities have you heard of before? Select all that you are familiar with | World Antimicrobial Awareness Week |
| Which of the following terms or activities have you heard of before? Select all that you are familiar with | None of the above |
| Which one of the following antibiotics may be safe during pregnancy? |  |
|  | Amoxicillin |
|  | Ciprofloxacin |
|  | Gentamicin |
|  | I don't know |
| Which one of the following antibiotics has the best activity against anaerobes? |  |
|  | Ciprofloxacin |
|  | Metronidazole |
|  | Trimethoprim-sulfamethoxazole |
|  | I don't know |
| Methicillin resistant-Staphylococcus aureus (MRSA) is susceptible to: |  |
|  | Cefalotin |
|  | Ceftriaxone |
|  | Cefuroxime |
|  | I don't know |
|  | None of these |
| Which one of the following antibiotics is more effective in crossing the blood-brain barrier? |  |
|  | Ceftriaxone |
|  | Clindamycin |
|  | Vancomycin |
|  | I don't know |
| Which of these conditions can be treated with antibiotics? Select all that apply | HIV/AIDS |
| Which of these conditions can be treated with antibiotics? Select all that apply | Gonorrhoea |
| Which of these conditions can be treated with antibiotics? Select all that apply | Bladder |
| Which of these conditions can be treated with antibiotics? Select all that apply | Diarrhoea |
| Which of these conditions can be treated with antibiotics? Select all that apply | Cold and flu |
| Which of these conditions can be treated with antibiotics? Select all that apply | Fever |
| Which of these conditions can be treated with antibiotics? Select all that apply | Malaria |
| Which of these conditions can be treated with antibiotics? Select all that apply | Measles |
| Which of these conditions can be treated with antibiotics? Select all that apply | Sore throat |
| Which of these conditions can be treated with antibiotics? Select all that apply | Body aches |
| Which of these conditions can be treated with antibiotics? Select all that apply | Skin or wound infection |
| Which of these conditions can be treated with antibiotics? Select all that apply | Headaches |
| Which of these conditions can be treated with antibiotics? Select all that apply | None of the above |
| Antibiotics are used to treat infections caused by bacteria. |  |
|  | TRUE |
|  | FALSE |
| Antibiotics are used to treat infections caused by viruses. |  |
|  | TRUE |
|  | FALSE |
| Antimicrobial resistance can be caused by the overuse and misuse of antimicrobials in humans. |  |
|  | TRUE |
|  | FALSE |
| Antimicrobial resistance can be caused by the overuse and misuse of antimicrobials in animals. |  |
|  | TRUE |
|  | FALSE |
| Antibiotic resistance can transfer between humans, animals, and the environment. |  |
|  | TRUE |
|  | FALSE |
| Antibiotic resistance occurs when bacteria and viruses stop responding to antimicrobials. |  |
|  | TRUE |
|  | FALSE |
| Healthy people can carry antibiotic-resistant bacteria. |  |
|  | TRUE |
|  | FALSE |
| Giving antimicrobials to people who are not sick will prevent them from becoming sick. |  |
|  | TRUE |
|  | FALSE |
| Many bacteria that cause illnesses are becoming increasingly resistant to treatment by antibiotics. |  |
|  | TRUE |
|  | FALSE |
| Antibiotic-resistant infections can cause death. |  |
|  | TRUE |
|  | FALSE |
| If bacteria are resistant to antibiotics, it can be very difficult or even impossible to treat the infections they cause. |  |
|  | TRUE |
|  | FALSE |
| Antibiotic resistance is only a problem for people who are given antibiotics frequently. |  |
|  | TRUE |
|  | FALSE |
| If you give patients antimicrobials too often, they will stop working. |  |
|  | TRUE |
|  | FALSE |
| Vaccination can reduce or slow antimicrobial resistance. |  |
|  | TRUE |
|  | FALSE |
| Good hygiene and sanitation practices can reduce or slow antimicrobial resistance. |  |
|  | TRUE |
|  | FALSE |
| Which of the following are classified as World Health Organization (WHO) “Reserve” drugs. | Macrolides (i.e. azithromycin) |
|  | Aztreonam |
|  | Polymyxins (i.e. colistin) |
|  | Ampicillin |
|  | 4th- generation cephalosporins (i.e. cefepime) |
|  | I don't know |
| How does the World Health Organization (WHO) define "Reserve" drugs? |  |
|  | Antibiotics with higher resistance potential that should used sparingly |
|  | Antimicrobials that can be used any time for first- or second-line treatment |
|  | Last-resort drugs that should only be used in specific cases or patient populations or when alternative treatment has failed. |
|  | I don't know |
| Do you know how to report suspicion of substandard or falsified medicinal products including antimicrobials in your country? |  |
|  | No |
|  | There is no reporting mechanism in my facility or country |
|  | Yes |
|  |  |
| **Attitudes** | |
|  | |
| **Ǫuestion** | **Options** |
| How confident are you that pharmacists use antimicrobials optimally? |  |
|  | Unconfident |
|  | Somewhat unconfident |
|  | Somewhat Confident |
|  | Very Confident |
| How confident are you that you use antimicrobials optimally? |  |
|  | Unconfident |
|  | Somewhat unconfident |
|  | Somewhat Confident |
|  | Very Confident |
| Antimicrobial resistance is a serious public health problem in my country. |  |
|  | Disagree Strongly |
|  | Disagree Slightly |
|  | Neither agree nor disagree |
|  | Agree slightly |
|  | Agree Strongly |
| In general, I feel antimicrobials are overused. |  |
|  | Disagree Strongly |
|  | Disagree Slightly |
|  | Neither agree nor disagree |
|  | Agree slightly |
|  | Agree Strongly |
| In the community, I think antimicrobials are overused through non-prescription consumption. |  |
|  | Disagree Strongly |
|  | Disagree Slightly |
|  | Neither agree nor disagree |
|  | Agree slightly |
|  | Agree Strongly |
| Antimicrobial resistance should be considered before prescribing antimicrobials. |  |
|  | Disagree Strongly |
|  | Disagree Slightly |
|  | Neither agree nor disagree |
|  | Agree slightly |
|  | Agree Strongly |
| Patients’ demands for antimicrobials contribute to their prescription. |  |
|  | Disagree Strongly |
|  | Disagree Slightly |
|  | Neither agree nor disagree |
|  | Agree slightly |
|  | Agree Strongly |
| More judicious use of antimicrobials would reduce resistance. |  |
|  | Disagree Strongly |
|  | Disagree Slightly |
|  | Neither agree nor disagree |
|  | Agree slightly |
|  | Agree Strongly |
| I suspect that some antibiotics available in my facility are of poor quality and for that reason do not work. |  |
|  | Disagree Strongly |
|  | Disagree Slightly |
|  | Neither agree nor disagree |
|  | Agree slightly |
|  | Agree Strongly |
| The unavailability of clinical diagnostic tools and tests impacts my ability to select, recommend, or prescribe antimicrobials for diseases. |  |
|  | Disagree Strongly |
|  | Disagree Slightly |
|  | Neither agree nor disagree |
|  | Agree slightly |
|  | Agree Strongly |
| Access to appropriate antimicrobials is a challenge in my facility because there are frequent stock-outs. |  |
|  | Disagree Strongly |
|  | Disagree Slightly |
|  | Neither agree nor disagree |
|  | Agree slightly |
|  | Agree Strongly |
| Even when I seek out laboratory testing, I do not always trust the results. |  |
|  | Disagree Strongly |
|  | Disagree Slightly |
|  | Neither agree nor disagree |
|  | Agree slightly |
|  | Agree Strongly |
| In my country, there are sufficient awareness and education activities on antimicrobial resistance and stewardship awareness. |  |
|  | Disagree Strongly |
|  | Disagree Slightly |
|  | Neither agree nor disagree |
|  | Agree slightly |
|  | Agree Strongly |
| In my facility, I think antimicrobials are overused. |  |
|  | Disagree Strongly |
|  | Disagree Slightly |
|  | Neither agree nor disagree |
|  | Agree slightly |
|  | Agree Strongly |
|  |  |
| Do you think antimicrobial stewardship (AMS) activities have any of the following impacts? Select all that apply | They improve the quality of medical care |
|  | They reduce the cost of treatment |
|  | They reduce the overuse of antimicrobials |
|  | They promote reasonable prescription of antimicrobials |
|  | They help control antimicrobial resistance |
|  | None of the above |
| **Practices** | |
| **Ǫuestion** | **Options** |
| Dispense or sell antimicrobials to a patient with a prescription from a licensed healthcare provide |  |
|  | More than five times daily |
|  | More than once daily |
|  | Daily |
|  | Once a week |
|  | Once a month |
|  | Less than once per month |
|  | I did not prescribe antimicrobials in the past six (6) months |
| Dispense or sell antimicrobials to a patient without a prescription? |  |
|  | More than five times daily |
|  | More than once daily |
|  | Daily |
|  | Once a week |
|  | Less than once per month |
|  | Once a month |
|  | I did not prescribe antimicrobials in the past six (6) months |
| Dispensed or sold an antimicrobial empirically (i.e. without clinically confirmed diagnosis or antimicrobial susceptibility testing [AST]) |  |
|  | Never |
|  | Rarely |
|  | Sometimes |
|  | Always |
| Had access to antimicrobial susceptibility testing (AST) |  |
|  | Never |
|  | Rarely |
|  | Sometimes |
|  | Always |
| Used antimicrobial susceptibility testing (AST) results to inform your decision on antimicrobial selection or prescription |  |
|  | Never |
|  | Rarely |
|  | Sometimes |
|  | Always |
| Dispensed or sold an antimicrobial based on recommendations of pharmaceutical companies |  |
|  | Never |
|  | Rarely |
|  | Sometimes |
|  | Always |
| Dispensed or sold an antimicrobial for malaria or suspected malaria |  |
|  | Never |
|  | Rarely |
|  | Sometimes |
|  | Always |
| Dispensed or sold an antimicrobial because the patient requested one |  |
|  | Never |
|  | Rarely |
|  | Sometimes |
|  | Always |
| Consulted a local, national, or international treatment guideline to inform clinical decision making around antimicrobial use or prescription |  |
|  | Never |
|  | Rarely |
|  | Sometimes |
|  | Always |
| Educated patients about prudent use of antimicrobials when you dispensed or sold them |  |
|  | Never |
|  | Rarely |
|  | Sometimes |
|  | Always |
| Educated patients on antibiotic resistance and its dangers due to misuse |  |
|  | Never |
|  | Rarely |
|  | Sometimes |
|  | Always |
| Encountered medicinal products which you suspected may be substandard or falsified |  |
|  | Never |
|  | Rarely |
|  | Sometimes |
|  | Always |
| Which of the following are available or have been implemented at your facility? Select all that apply | Antimicrobial stewardship committee |
|  | Facility/local treatment guidelines |
|  | National treatment guidelines |
|  | International treatment guidelines |
|  | Approval |
|  | Facility antibiogram or resistance data |
|  | National antibiogram or resistance data |
|  | Regular feedbac |
|  | Grand rounds with antimicrobial use and stewardship focus |
|  | Other |
|  | None/Not apllicable |
| Do you participate in antimicrobial stewardship activities in your facility? |  |
|  | Yes |
|  | No |
|  | There are no such activities at my facility. |
| Why not? |  |
|  | I have limited knowledge of stewardship. |
|  | It won’t make much difference |
|  | Lack of time due to other work. |
|  | The facility where I work does not have any antimicrobial |
|  | It is the healthcare provider’s domain |
|  | Other |
| Which of the following procedures or interventions do you think would be most helpful in ensuring appropriate antimicrobial use? Select all that apply | Antimicrobial stewardship committee |
|  | Facility/local treatment guidelines |
|  | National treatment guidelines |
|  | International treatment guidelines |
|  | Approval/review process for use of restricted antibiotics |
|  | Facility antibiogram or resistance data |
|  | National antibiogram or resistance data |
|  | Regular feedback |
|  | Grand rounds with antimicrobial use and stewardship focus |
|  | Other |
|  | None |
| Which of the following sources of information do you use as part of continuous medical education on antibiotics or when you have a specific question on antibiotics | Colleagues |
|  | Internet |
|  | Locally developed treatment guidelines |
|  | National treatment guidelines |
|  | Social media |
|  | Other |
|  | Mobile phone to search online |
|  | Antimicrobial prescribing application |
|  |  |
| **Training and education** | |
|  |  |
| **Ǫuestion** | **Options** |
| Have you ever had formal training or education on antimicrobial use, resistance, or stewardship | Yes- as part of my formal education |
|  | Yes- as part of my on-the-job training |
|  | Yes- through extracurricular activities, personal interest, or career development |
|  | No |
| What kind of education or training have you received? Select all that apply | Dedicated course |
|  | Lecture within a course |
|  | Extracurricular |
|  | Grand rounds |
|  | Other |
| In the past year, how often did you receive formal training or education on antimicrobial use, stewardship, or resistance? |  |
|  | Monthly or more frequently |
|  | Monthly |
|  | Ǫuarterly |
|  | Once during the year |
|  | I did not receive any training or education in the past year |
| Who developed or organized the training? |  |
|  | International organisation or body |
|  | Local facility or health department |
|  | Local government |
|  | Local or national non-governmental organization (NGO) |
|  | National government |
|  | Online materials |
|  | Other |
|  | Pharmacy school |
|  | Professional Association (PSK and PPB) |
|  | Professional Association (PSK) |
| Do you think staff at your facility would benefit from additional training an.or education on antimicrobial use, resistance, an.or stewardship? |  |
|  | No |
|  | Yes |
| Do you think you have enough sources of information about antibiotics when you need it? |  |
|  | No |
|  | Yes |

## Supplementary Table 2. List of countries included in the survey (Regions based on the classification of global burden of disease)

### Supplementary Table 2.1 List of countries with fewer than five responders

| **Country** | **Region** | **N (number of respondents)** |
| --- | --- | --- |
| Tunisia | North Africa and Middle East | 1 |
| Cameroon | Western sub-Saharan Africa | 4 |
| Gambia | Western sub-Saharan Africa | 3 |
| Liberia | Western sub-Saharan Africa | 1 |
| Niger | Western sub-Saharan Africa | 1 |
| Comoros | Eastern sub-Saharan Africa | 1 |
| South Sudan | Eastern sub-Saharan Africa | 4 |
| Eswatini | Southern sub-Saharan Africa | 4 |
| Malawi | Southern sub-Saharan Africa | 4 |
| Namibia | Southern sub-Saharan Africa | 3 |

### Supplementary Table 2.2. List of countries with more than five responders

| **Country** | **Region** | **N (number of respondents)** |
| --- | --- | --- |
| Egypt | North Africa and Middle East | 101 |
| Burkina Faso | Western sub-Saharan Africa | 7 |
| Ghana | Western sub-Saharan Africa | 187 |
| Nigeria | Western sub-Saharan Africa | 70 |
| Sierra Leone | Western sub-Saharan Africa | 7 |
| Togo | Western sub-Saharan Africa | 16 |
| Burundi | Eastern sub-Saharan Africa | 11 |
| Ethiopia | Eastern sub-Saharan Africa | 60 |
| Kenya | Eastern sub-Saharan Africa | 115 |
| Rwanda | Eastern sub-Saharan Africa | 6 |
| Somalia | Eastern sub-Saharan Africa | 18 |
| Sudan | Eastern sub-Saharan Africa | 23 |
| Tanzania | Eastern sub-Saharan Africa | 29 |
| Uganda | Eastern sub-Saharan Africa | 117 |
| Zambia | Eastern sub-Saharan Africa | 59 |
| Lesotho | Southern sub-Saharan Africa | 9 |
| South Africa | Southern sub-Saharan Africa | 28 |
| Zimbabwe | Southern sub-Saharan Africa | 19 |

## Supplementary Table 3: Knowledge questions and responses scored and analysed in logistic regression

### Supplementary Table 3.1: True/False questions (Q36-Q50)

| **Knowledge Questions** | **Correct Answer** | **Correct (%)** | **Incorrect (%)** |
| --- | --- | --- | --- |
| antibiotics are used to treat infections caused by bacteria. | true | 878 (96.7) | 30 (3.3) |
| antibiotics are used to treat infections caused by viruses. | false | 669 (73.68) | 239 (26.32) |
| antimicrobial resistance can be caused by the overuse and misuse of antimicrobials in humans. | true | 815 (89.76) | 93 (10.24) |
| antimicrobial resistance can be caused by the overuse and misuse of antimicrobials in animals. | true | 686 (75.55) | 222 (24.45) |
| antibiotic resistance can transfer between humans, animals, and the environment. | true | 631 (69.49) | 277 (30.51) |
| antibiotic resistance occurs when bacteria and viruses stop responding to antimicrobials. | true | 641 (70.59) | 267 (29.41) |
| healthy people can carry antibiotic-resistant bacteria. | true | 734 (80.84) | 174 (19.16) |
| giving antimicrobials to people who are not sick will prevent them from becoming sick. | true | 249 (27.42) | 659 (72.58) |
| many bacteria that cause illnesses are becoming increasingly resistant to treatment by antibiotics. | true | 801 (88.22) | 107 (11.78) |
| antibiotic-resistant infections can cause death. | true | 790 (87) | 118 (13) |
| if bacteria are resistant to antibiotics, it can be very difficult or even impossible to treat the infections they cause. | true | 827 (91.08) | 81 (8.92) |
| antibiotic resistance is only a problem for people who are given antibiotics frequently. | false | 480 (52.86) | 428 (47.14) |
| if you give patients antimicrobials too often, they will stop working. | true | 652 (71.81) | 256 (28.19) |
| vaccination can reduce or slow antimicrobial resistance. | true | 547 (60.24) | 361 (39.76) |
| good hygiene and sanitation practices can reduce or slow antimicrobial resistance. | true | 722 (79.52) | 186 (20.48) |

### Supplementary Table 3.2 Multiple choice knowledge questions (Q23-Q35)

| **Knowledge Question** | **Correct Answer** | **Correct (%)** | **Incorrect (%)** |
| --- | --- | --- | --- |
| which of these conditions can be treated with antibiotics? select all that apply. | not hiv/aids | 548 (60.35) | 360 (39.65) |
|  | gonorrhoea | 550 (60.57) | 358 (39.43) |
|  | bladder infection or urinary tract infection | 635 (69.93) | 273 (30.07) |
|  | diarrhoea | 343 (37.78) | 565 (62.22) |
|  | not cold and flu | 710 (78.19) | 198 (21.81) |
|  | not fever | 742 (81.72) | 166 (18.28) |
|  | not malaria | 722 (79.52) | 186 (20.48) |
|  | not measles | 800 (88.11) | 108 (11.89) |
|  | not sore throat | 558 (61.45) | 350 (38.55) |
|  | not body aches | 793 (87.33) | 115 (12.67) |
|  | skin or wound infection | 589 (64.87) | 319 (35.13) |
|  | not headaches | 832 (91.63) | 76 (8.37) |
|  | not none of the above | 907 (99.89) | 1 (0.11) |

## Supplementary Table 4: Attitude questions and responses

| **Question** | **Responses** | **N (%)** |
| --- | --- | --- |
| How confident are you that pharmacists use antimicrobials optimally? |  |  |
|  | Unconfident | 28 (3.1) |
|  | Somewhat unconfident | 100 (11.0) |
|  | Somewhat Confident | 367 (40.4) |
|  | Very Confident | 407 (44.8) |
| How confident are you that you use antimicrobials optimally? |  |  |
|  | Unconfident | 16 (1.8) |
|  | Somewhat unconfident | 56 (6.2) |
|  | Somewhat Confident | 344 (37.9) |
|  | Very Confident | 485 (53.4) |
| Antimicrobial resistance is a serious public health problem in my country.***** |  |  |
|  | Disagree Strongly | 10 (1.1) |
|  | Disagree Slightly | 33 (3.6) |
|  | Neither agree nor disagree | 69 (7.6) |
|  | Agree slightly | 232 (25.6) |
|  | Agree Strongly | 555 (61.1) |
| In general, I feel antimicrobials are overused. |  |  |
|  | Disagree Strongly | 10 (1.1) |
|  | Disagree Slightly | 26 (2.9) |
|  | Neither agree nor disagree | 71 (7.8) |
|  | Agree slightly | 250 (27.5) |
|  | Agree Strongly | 540 (59.5) |
| In the community, I think antimicrobials are overused through non-prescription consumption.***** |  |  |
|  | Disagree Strongly | 19 (2.1) |
|  | Disagree Slightly | 33 (3.6) |
|  | Neither agree nor disagree | 91 (10.0) |
|  | Agree slightly | 240 (26.4) |
|  | Agree Strongly | 515 (56.7) |
| Antimicrobial resistance should be considered before prescribing antimicrobials.***** |  |  |
|  | Disagree Strongly | 12 (1.3) |
|  | Disagree Slightly | 15 (1.7) |
|  | Neither agree nor disagree | 53 (5.8) |
|  | Agree slightly | 194 (21.4) |
|  | Agree Strongly | 622 (68.5) |
| Patients’ demands for antimicrobials contribute to their prescription.***** |  |  |
|  | Disagree Strongly | 51 (5.6) |
|  | Disagree Slightly | 69 (7.6) |
|  | Neither agree nor disagree | 121 (13.3) |
|  | Agree slightly | 315 (34.7) |
|  | Agree Strongly | 342 (37.7) |
| More judicious use of antimicrobials would reduce resistance.***** |  |  |
|  | Disagree Strongly | 19 (2.1) |
|  | Disagree Slightly | 37 (4.1) |
|  | Neither agree nor disagree | 109 (12.0) |
|  | Agree slightly | 188 (20.7) |
|  | Agree Strongly | 541 (59.6) |
| I suspect that some antibiotics available in my facility are of poor quality and for that reason do not work. |  |  |
|  | Disagree Strongly | 143 (15.7) |
|  | Disagree Slightly | 163 (18.0) |
|  | Neither agree nor disagree | 173 (19.1) |
|  | Agree slightly | 221 (24.3) |
|  | Agree Strongly | 197 (21.7) |
| The unavailability of clinical diagnostic tools and tests impacts my ability to select, recommend, or prescribe antimicrobials for diseases.***** |  |  |
|  | Disagree Strongly | 17 (1.9) |
|  | Disagree Slightly | 17 (1.9) |
|  | Neither agree nor disagree | 35 (3.9) |
|  | Agree slightly | 71 (7.8) |
|  | Agree Strongly | 155 (17.1) |
| Access to appropriate antimicrobials is a challenge in my facility because there are frequent stock-outs.***** |  |  |
|  | Disagree Strongly | 99 (10.9) |
|  | Disagree Slightly | 130 (14.3) |
|  | Neither agree nor disagree | 149 (16.4) |
|  | Agree slightly | 273 (30.1) |
|  | Agree Strongly | 244 (26.9) |
| Even when I seek out laboratory testing, I do not always trust the results.***** |  |  |
|  | Disagree Strongly | 84 (9.3) |
|  | Disagree Slightly | 66 (7.3) |
|  | Neither agree nor disagree | 69 (7.6) |
|  | Agree slightly | 49 (5.4) |
|  | Agree Strongly | 26 (2.9) |
| In my country, there are sufficient awareness and education activities on antimicrobial resistance and stewardship awareness. |  |  |
|  | Disagree Strongly | 139 (15.3) |
|  | Disagree Slightly | 162 (17.8) |
|  | Neither agree nor disagree | 138 (15.2) |
|  | Agree slightly | 240 (26.4) |
|  | Agree Strongly | 219 (24.1) |
| In my facility, I think antimicrobials are overused.***** |  |  |
|  | Disagree Strongly | 90 (9.9) |
|  | Disagree Slightly | 135 (14.9) |
|  | Neither agree nor disagree | 172 (18.9) |
|  | Agree slightly | 253 (27.9) |
|  | Agree Strongly | 245 (27.0) |
| Do you think antimicrobial stewardship (AMS) activities have any of the following impacts? Select all that apply. | They improve the quality of medical care. | 689 (75.9) |
|  | They reduce the cost of treatment. | 461 (50.8) |
|  | They reduce the overuse of antimicrobials. | 611 (67.3) |
|  | They promote reasonable prescription of antimicrobials. | 559 (61.6) |
|  | They help control antimicrobial resistance. | 524 (57.7) |
|  | None of the above | 202 (22.2) |

The 9 questions ending with an asterisk sign (*) were used in scoring the participants attitudes towards AMR.

## Supplementary Table 5: Practice questions and responses.

| Questions | Responses | N (%) |
| --- | --- | --- |
| Dispense or sell antimicrobials to a patient with a prescription from a licensed healthcare provide. |  |  |
|  | More than five times daily | 122 (13.4) |
|  | More than once daily | 115 (12.7) |
|  | Daily | 246 (27.1) |
|  | Once a week | 124 (13.7) |
|  | Once a month | 116 (12.8) |
|  | Less than once per month | 110 (12.1) |
|  | I did not prescribe antimicrobials in the past six (6) months. | 52 (5.7) |
| Dispense or sell antimicrobials to a patient without a prescription?***** |  |  |
|  | More than five times daily | 32 (3.5) |
|  | More than once daily | 69 (7.6) |
|  | Daily | 146 (16.1) |
|  | Once a week | 132 (14.5) |
|  | Less than once per month | 118 (13.0) |
|  | Once a month | 244 (26.9) |
|  | I did not prescribe antimicrobials in the past six (6) months. | 128 (14.1) |
| Dispensed or sold an antimicrobial empirically (i.e. without clinically confirmed diagnosis or antimicrobial susceptibility testing [AST]) |  |  |
|  | Never | 315 (34.7) |
|  | Rarely | 221 (24.3) |
|  | Sometimes | 259 (28.5) |
|  | Always | 98 (10.8) |
| Had access to antimicrobial susceptibility testing (AST)***** |  |  |
|  | Never | 272 (30.0) |
|  | Rarely | 251 (27.6) |
|  | Sometimes | 290 (31.9) |
|  | Always | 82 (9.0) |
| Used antimicrobial susceptibility testing (AST) results to inform your decision on antimicrobial selection or prescription |  |  |
|  | Never | 252 (27.8) |
|  | Rarely | 220 (24.2) |
|  | Sometimes | 273 (30.1) |
|  | Always | 146 (16.1) |
| Dispensed or sold an antimicrobial based on recommendations of pharmaceutical companies.***** |  |  |
|  | Never | 230 (25.3) |
|  | Rarely | 195 (21.5) |
|  | Sometimes | 301 (33.1) |
|  | Always | 166 (18.3) |
| Dispensed or sold an antimicrobial for malaria or suspected malaria.***** |  |  |
|  | Never | 363 (40.0) |
|  | Rarely | 150 (16.5) |
|  | Sometimes | 241 (26.5) |
|  | Always | 134 (14.8) |
| Dispensed or sold an antimicrobial because the patient requested one.***** | Q65 |  |
|  | Never | 341 (37.6) |
|  | Rarely | 199 (21.9) |
|  | Sometimes | 280 (30.8) |
|  | Always | 73 (8.0) |
| Consulted a local, national, or international treatment guideline to inform clinical decision making around antimicrobial use or prescription.***** |  |  |
|  | Never | 120 (13.2) |
|  | Rarely | 158 (17.4) |
|  | Sometimes | 358 (39.4) |
|  | Always | 254 (28.0) |
| Educated patients about prudent use of antimicrobials when you dispensed or sold them.***** |  |  |
|  | Never | 86 (9.5) |
|  | Rarely | 94 (10.4) |
|  | Sometimes | 269 (29.6) |
|  | Always | 445 (49.0) |
| Educated patients on antibiotic resistance and its dangers due to misuse.***** |  |  |
|  | Never | 84 (9.3) |
|  | Rarely | 93 (10.2) |
|  | Sometimes | 288 (31.7) |
|  | Always | 429 (47.2) |
| Encountered medicinal products which you suspected may be substandard or falsified.***** |  |  |
|  | Never | 73 (8.0) |
|  | Rarely | 81 (8.9) |
|  | Sometimes | 76 (8.4) |
|  | Always | 25 (2.8) |
| Which of the following are available or have been implemented at your facility? Select all that apply. | Antimicrobial stewardship committee | 442 (48.7) |
|  | Facility/local treatment guidelines | 479 (52.8) |
|  | National treatment guidelines | 445 (49.0) |
|  | International treatment guidelines | 283 (31.2) |
|  | Approval/review process for use of restricted antibiotics | 192 (21.1) |
|  | Facility antibiogram or resistance data | 186 (20.5) |
|  | National antibiogram or resistance data | 118 (13.0) |
|  | Regular feedback/audit on individual prescribing practices | 145 (16.0) |
|  | Grand rounds with antimicrobial use and stewardship focus | 157 (17.3) |
|  | Other | 19 (2.1) |
|  | None/Not Applicable | 57 (6.3) |
| Do you participate in antimicrobial stewardship activities in your facility? |  |  |
|  | Yes | 523 (57.6) |
|  | No | 187 (20.6) |
|  | There are no such activities at my facility. | 180 (19.8) |
|  | Missing | 18 (2.0) |
| Why not? |  |  |
|  | I have limited knowledge of stewardship. | 43 (4.7) |
|  | It won’t make much difference | 10 (1.1) |
|  | Lack of time due to other work. | 59 (6.5) |
|  | The facility where I work does not have any antimicrobial stewardship activities | 56 (6.2) |
|  | It is the healthcare provider’s domain | 27 (3.0) |
|  | Other | 1 (0.1) |
| Which of the following procedures or interventions do you think would be most helpful in ensuring appropriate antimicrobial use? Select all that apply. | Antimicrobial stewardship committee | 601 (66.2) |
|  | Facility/local treatment guidelines | 522 (57.5) |
|  | National treatment guidelines | 587 (64.6) |
|  | International treatment guidelines | 415 (45.7) |
|  | Approval/review process for use of restricted antibiotics | 433 (47.7) |
|  | Facility antibiogram or resistance data | 372 (41.0) |
|  | National antibiogram or resistance data | 342 (37.7) |
|  | Regular feedback/audit on individual prescribing practices | 411 (45.3) |
|  | Grand rounds with antimicrobial use and stewardship focus | 341 (37.6) |
|  | Other | 21 (2.3) |
|  | None | 6 (0.7) |
| Which of the following sources of information do you use as part of continuous medical education on antibiotics or when you have a specific question on antibiotics? | Colleagues | 510 (56.2) |
|  | Internet | 695 (76.5) |
|  | Locally developed treatment guidelines | 353 (38.9) |
|  | National treatment guidelines | 558 (61.5) |
|  | Social media | 194 (21.4) |
|  | Other | 24 (2.6) |
|  | Mobile phone to search online | 132 (14.5) |
|  | Antimicrobial prescribing application | 60 (6.6) |

The nine questions ending with an asterisk sign (*) were used in scoring the AMR practices.

## Supplementary Table 6: Spearman’s correlation matrix

|  | **Age** | Years of experience | Job Title | Gender | Job Setting |
| --- | --- | --- | --- | --- | --- |
| Age | 1 | 0.616 | 0.068 | -0.037 | 0.087 |
| Years of experience | 0.616 | 1 | -0.022 | 0.004 | 0.074 |
| Job Title | 0.068 | -0.022 | 1 | 0.006 | -0.007 |
| Gender | -0.037 | 0.004 | 0.006 | 1 | 0.098 |
| Job Setting | 0.087 | 0.074 | -0.007 | 0.098 | 1 |

## Supplementary Table 7: Univariate logistic regression

### Supplementary Table 7.1 Univariate regression analysis of knowledge

| **Variable (N)** | **Good Knowledge**  **N (%)** | **aOR (95%CI)** | **P value** |
| --- | --- | --- | --- |
| **Gender** |  |  |  |
| Female (359) | 258 (71.90) | Ref. | 0.7421 |
| Male (549) | 389 (70.90) | 0.95 (0.7 - 1.30) |  |
| **Years of Experience** |  |  |  |
| ≤1 (139) | 89 (64.00) | Ref. | <0.001 |
| 2_5 (423) | 285 (67.40) | 1.16 (0.78 - 1.72) |  |
| 6_10 (184) | 137 (74.50) | 1.64 (0.93 - 2.88) |  |
| 11_15 (82) | 69 (84.10) | 2.98 (1.66 - 5.36) |  |
| >16 (80) | 67 (83.80) | 2.9 (1.6 - 5.23) |  |
| **Job title** |  |  |  |
| Pharmacy technician (213) | 133 (62.40) | Ref. | <0.001 |
| Drug store owner or staff (114) | 77(67.50) | 1.25 (0.88 - 1.77) |  |
| Licensed Pharmacist (426) | 329(77.20) | 2.04 (1.43 - 2.92) |  |
| Pharmacy manager (155) | 108(69.70) | 1.38 (0.93 - 2.06) |  |
| **Job Setting** |  |  |  |
| Independent private pharmacy (334) | 229(68.60) | Ref. | <0.001 |
| Chain private pharmacy, medical store, or dispensary (93) | 60(64.50) | 0.83 (0.55 - 1.27) |  |
| Multiple facilities (47) | 44(93.60) | 6.72 (1.92 - 23.55) |  |
| Pharmacy or dispensary within a public health facility (153) | 110(71.90) | 1.17 (0.63 - 2.18) |  |
| Pharmacy or dispensary within a private/FBO/NGO health facility (181) | 131(72.40) | 1.2 (0.90 - 1.61) |  |
| University/ professional body/central store (57) | 42(73.70) | 1.28 (0.85 - 1.94) |  |
| Other (43) | 31(72.10) | 1.18 (0.63 - 2.21) |  |

N, number of responders

aOR, adjusted Odds Ratio

Ref., reference

### Supplementary Table 7.2 Univariate Regression Analysis of Attitude Scores

| **Variable (N)** | **Positive Attitudes**  **N (%)** | **aOR (95%CI)** | **P value** |
| --- | --- | --- | --- |
| **Gender** |  |  |  |
| Female (359) | 230(64.10) | Ref. | 0.0383 |
| Male (549) | 314(57.20) | 0.75 (0.59 - 0.96) |  |
| **Years of experience** |  |  |  |
| ≤1 (139) | 84 (60.40) | Ref. | <0.001 |
| 2_5 (423) | 224 (53.00) | 0.74 (0.5 - 1.09) |  |
| 6_10 (184) | 105 (57.10) | 0.87 (0.35 - 2.15) |  |
| 11_15 (82) | 68 (82.90) | 3.18 (2.21 - 4.57) |  |
| >16 (80) | 63 (78.80) | 2.43 (0.83 - 7.08) |  |
| **Job title** |  |  |  |
| Pharmacy technician (213) | 121 (56.80) | Ref. | <0.0001 |
| Drug store owner or staff (114) | 55 (48.20) | 0.71 (0.41 - 1.24) |  |
| Licensed Pharmacist (426) | 276 (64.80) | 1.4 (0.92 - 2.12) |  |
| Pharmacy manager (155) | 92 (59.40) | 1.11 (0.62 - 1.99) |  |
| **Job Setting** |  | Ref. |  |
| Independent private pharmacy (334) | 178 (53.30) | Ref. | <0.0001 |
| Chain private pharmacy, medical store, or dispensary (93) | 36 (38.70) | 0.55 (0.41 - 0.75) |  |
| Multiple facilities (47) | 40 (85.10) | 5.01 (2.6 - 9.65) |  |
| Pharmacy or dispensary within a public health facility (153) | 113 (73.90) | 2.48 (1.14 - 5.38) |  |
| Pharmacy or dispensary within a private/FBO/NGO health facility (181) | 107 (59.10) | 1.27 (0.74 - 2.18) |  |
| University/ Professional body/Central store (57) | 42 (73.70) | 2.45 (1.19 - 5.05) |  |
| Other (43) | 28 (65.10) | 1.64 (0.99 - 2.71) |  |

N, number of responders

aOR, adjusted Odds Ratio

Ref., reference

FBO, Faith-Based Organization

NGO, Non-Governmental Organization

### Supplementary Table 7.3 Univariate regression analysis of Practice Scores

| **Variable (N)** | **Positive Practice**  **N (%)** | **aOR (95%CI)** |  |
| --- | --- | --- | --- |
| **Gender** |  |  |  |
| Female (359) | 163 (45.40) | Ref. | 0.0624 |
| Male (549) | 215 (39.20) | 0.77 (0.61 - 0.98) |  |
| **Years of experience** |  |  |  |
| ≤1 (139) | 52 (37.40) | Ref. | 0.0001 |
| 2_5 (423) | 165 (39.00) | 1.07 (0.86 - 1.33) |  |
| 6_10 (184) | 72 (39.10) | 1.08 (0.89 - 1.30) |  |
| 11_15 (82) | 43 (52.40) | 1.84 (1.41 - 2.41) |  |
| >16 (80) | 46 (57.50) | 2.26 (0.74 - 6.9) |  |
| **Job title** |  |  |  |
| Pharmacy technician (213) | 94 (44.10) | Ref. | 0.007 |
| Drug store owner or staff (114) | 39 (34.20) | 0.66 (0.5 - 0.87) |  |
| Licensed Pharmacist (426) | 169 (39.70) | 0.83 (0.49 - 1.41) |  |
| Pharmacy manager (155) | 76 (49.00) | 1.22 (0.66 - 2.23) |  |
| **Job setting** |  |  |  |
| Independent private pharmacy (334) | 115 (34.40) | Ref. | <0.0001 |
| Chain private pharmacy, medical store, or dispensary (93) | 36 (38.70) | 1.2 (0.69 - 2.10) |  |
| Multiple facilities (47) | 19 (40.40) | 1.29 (0.46 - 3.63) |  |
| Pharmacy or dispensary within a public health facility (153) | 92 (60.10) | 2.87 (1.55 - 5.31) |  |
| Pharmacy or dispensary within a private/FBO/NGO health facility (181) | 76 (42.00) | 1.38 (1.02 - 1.87) |  |
| University/ professional body/central store (57) | 18(31.60) | 0.88 (0.53 - 1.45) |  |
| Other (43) | 22(51.20) | 2 (1.25 - 3.20) |  |

N, number of responders

aOR, adjusted Odds Ratio

Ref., reference

FBO, Faith-Based Organization

NGO, Non-Governmental Organization

## Model metrics

**
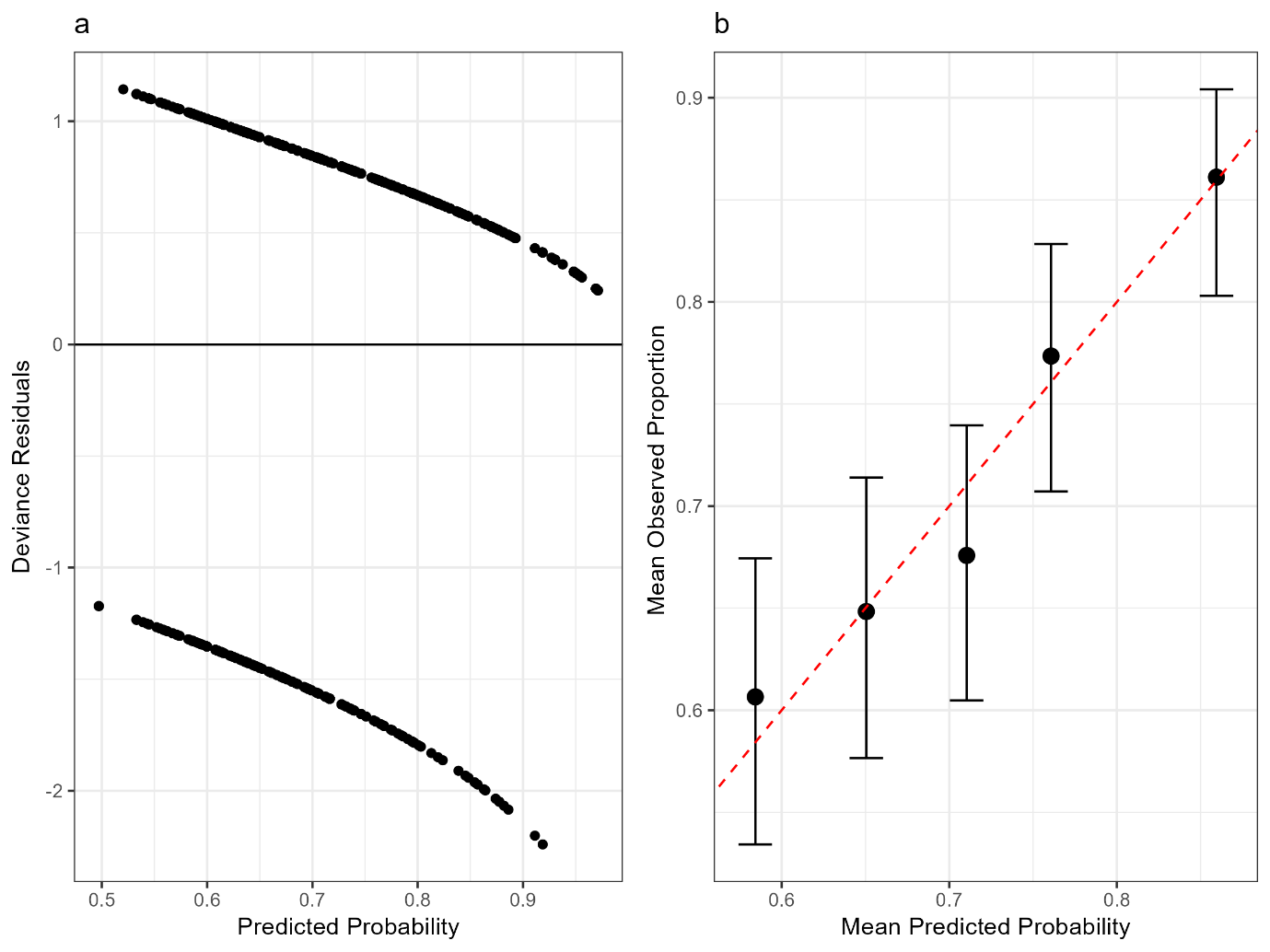
**

### Figure S1: Knowledge. Residuals plot (a) and calibration plot (b) for the AMR knowledge multivariable logistic regression model. The error bars on the calibration plot represent the 95% confidence interval (CI).

Goodness of fit statistics

χ² = 13.0, P = 0.112


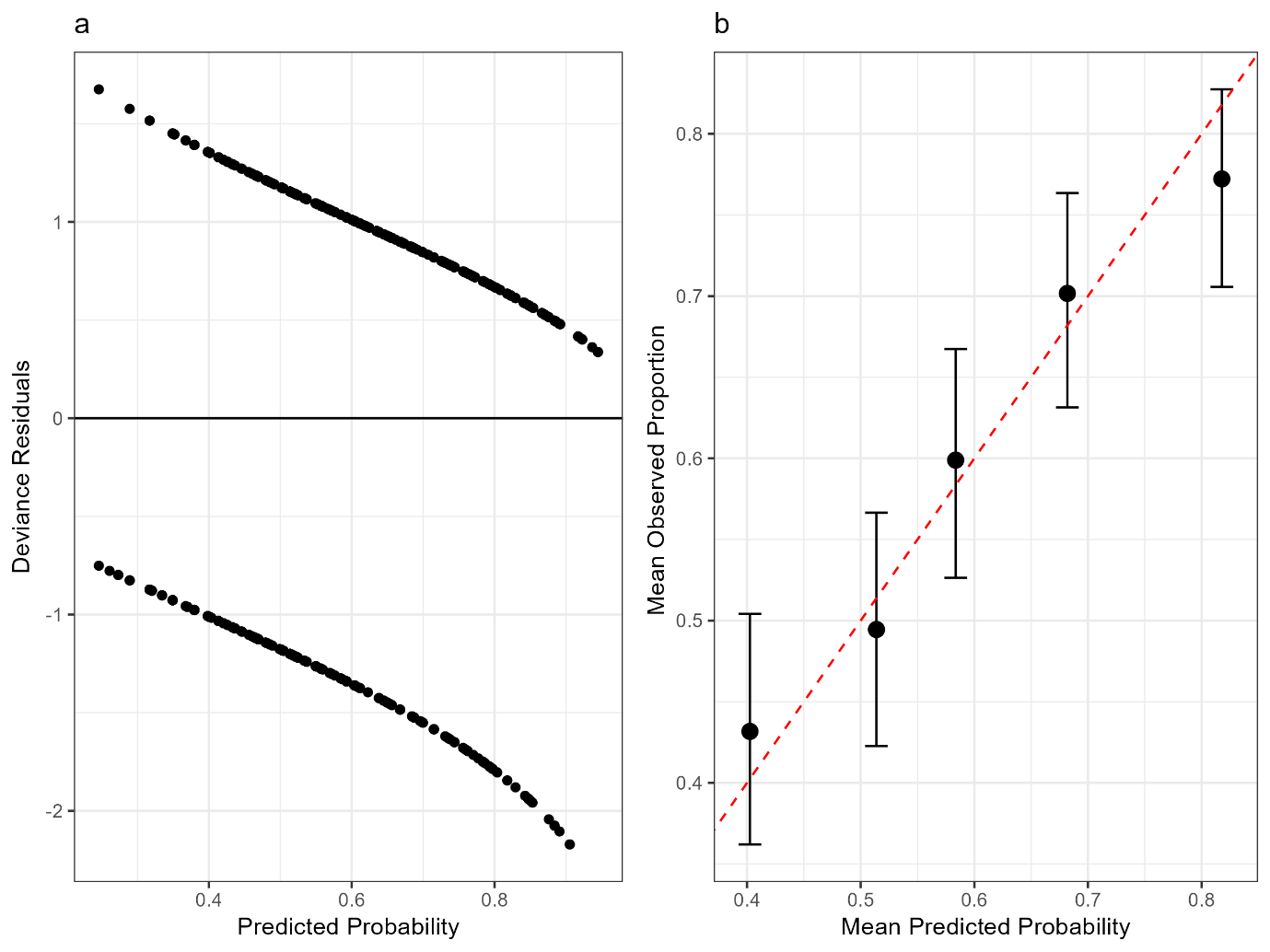


### Figure S2: Attitude. Residuals plot (a) and calibration plot (b) for the AMR attitude multivariable logistic regression model. The error bars on the calibration plot represent 95% CI.

Goodness of fit statistics

χ² = 6.26, P = 0.619

**
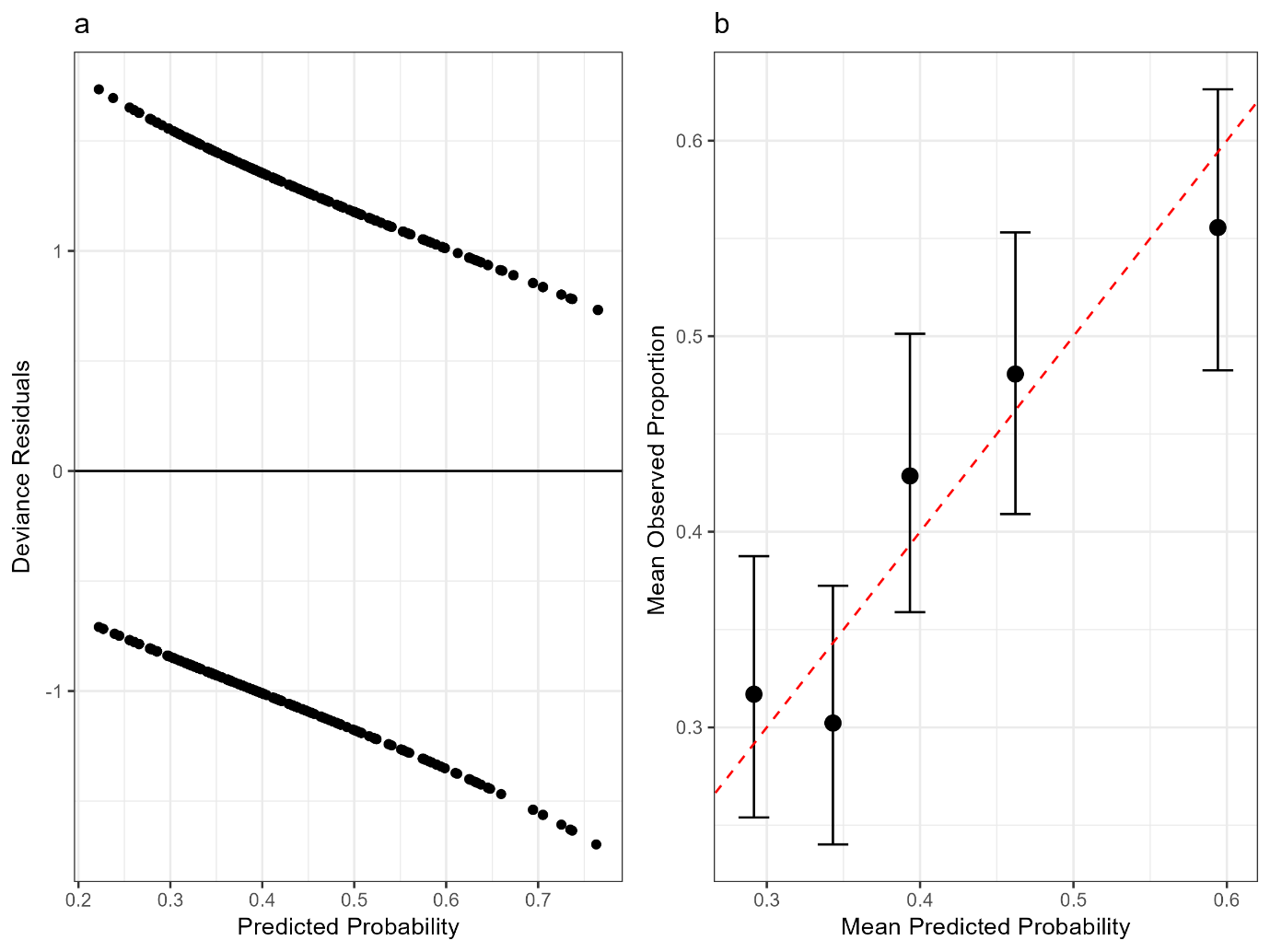
**

### Figure S3: Practice. Residuals plot (a) and calibration plot (b) for the AMR practices multivariable logistic regression model. The error bars on the calibration plot represent 95% CI.

Goodness of fit

χ² = 6.73, P = 0.566

## Supplementary Table 8: Distribution of knowledge, attitude, and practice AMR scores across Sub-Saharan Africa (SSA) regions

|  | **Percentage Mean (95% CI)** | | | | **ANOVA** | |
| --- | --- | --- | --- | --- | --- | --- |
| **Attribute** | **Northern SSA Africa (N=125)** | **Southern SSA Africa (N=63)** | **Western SSA Africa (296)** | **Eastern SSA Africa (N=424)** | **F-statistic** | **P value** |
| Knowledge | 73.1 (71.0-75.1) | 80.3 (77.3-83.3) | 73.2 (71.9-74.4) | 76.6 (75.5-77.6) | 11.506 | <0.001 |
| Attitude | 68.9 (66.3-71.5) | 82.0 (78.9-85.0) | 73.4 (71.6-75.1) | 76.3 (74.9-77.7) | 14.402 | <0.001 |
| Practice | 67.8 (66.0-69.7) | 72.5 (70.0-75.1) | 68.3 (67.1-69.6) | 69.7 (68.6-70.7) | 3.584 | 0.01 |

## Supplementary Table 9: Pairwise Comparisons of knowledge, attitude, and practice mean scores across African regions: Tukey's Honestly Significant Difference (HSD) test

| **Group1** | **Group2** | **mean difference (95% CI)** | **adjusted P value** |
| --- | --- | --- | --- |
| **Knowledge** | | | |
| Northern SSA | Eastern SSA | -3.52 (-6.42, -0.62) | 0.010 |
| Southern SSA | Eastern SSA | 3.73 (-0.11, 7.58) | 0.061 |
| Western SSA | Eastern SSA | -3.39 (-5.55, -1.24) | <0.001 |
| Southern SSA | Northern SSA | 7.25 (2.85, 11.65) | <0.001 |
| Western SSA | Northern SSA | 0.13 (-2.91, 3.16) | 1.000 |
| Western SSA | Southern SSA | -7.13 (-11.08, -3.18) | <0.001 |
| **Attitude** | | | |
| Northern SSA | Eastern SSA | -7.38 (-11.17, -3.58) | <0.001 |
| Southern SSA | Eastern SSA | 5.65 (0.61, 10.69) | 0.021 |
| Western SSA | Eastern SSA | -2.93 (-5.75, -0.1) | 0.039 |
| Southern SSA | Northern SSA | 13.02 (7.26, 18.79) | <0.001 |
| Western SSA | Northern SSA | 4.45 (0.47, 8.43) | 0.021 |
| Western SSA | Southern SSA | -8.57 (-13.75, -3.4) | <0.001 |
| **Practice** | | | |
| Northern SSA | Eastern SSA | -1.81 (-4.62, 1) | 0.348 |
| Southern SSA | Eastern SSA | 2.88 (-0.86, 6.61) | 0.195 |
| Western SSA | Eastern SSA | -1.33 (-3.42, 0.77) | 0.362 |
| Southern SSA | Northern SSA | 4.69 (0.41, 8.96) | 0.025 |
| Western SSA | Northern SSA | 0.48 (-2.47, 3.43) | 0.975 |
| Western SSA | Southern SSA | -4.2 (-8.04, -0.37) | 0.025 |

SSA: Sub-Saharan Africa

## Pairwise plots of Mean Differences in AMR Knowledge, attitude, and practice scores across African regions.

## (The reported mean difference is calculated by subtracting the mean of the second group from that of the first (e.g., for Southern vs. Western SSA, the mean for Western SSA is subtracted from the mean for Southern SSA)


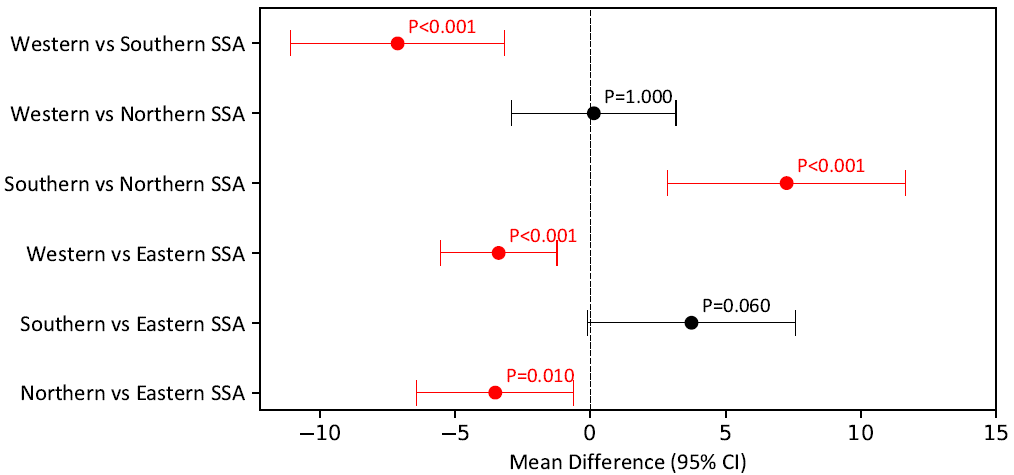


### Figure S4: Knowledge

**
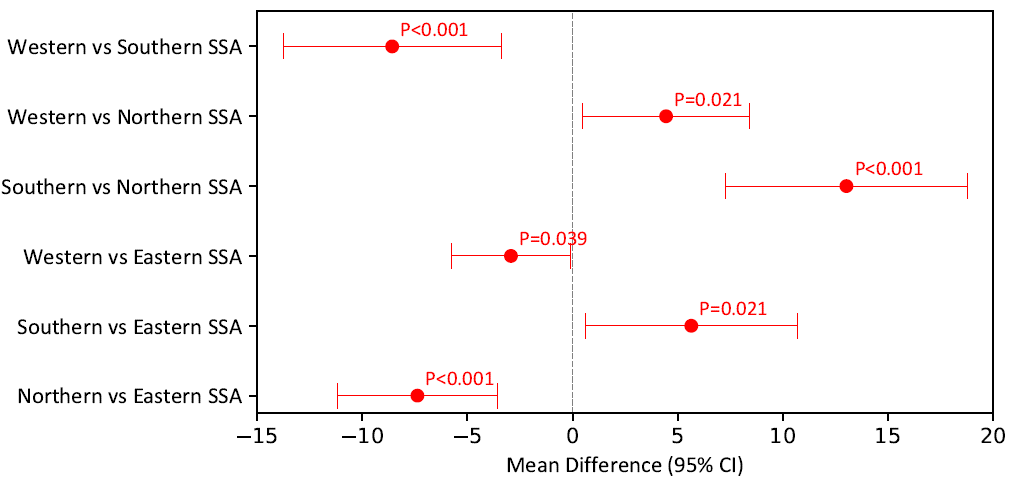
**

### Figure S5: Attitude


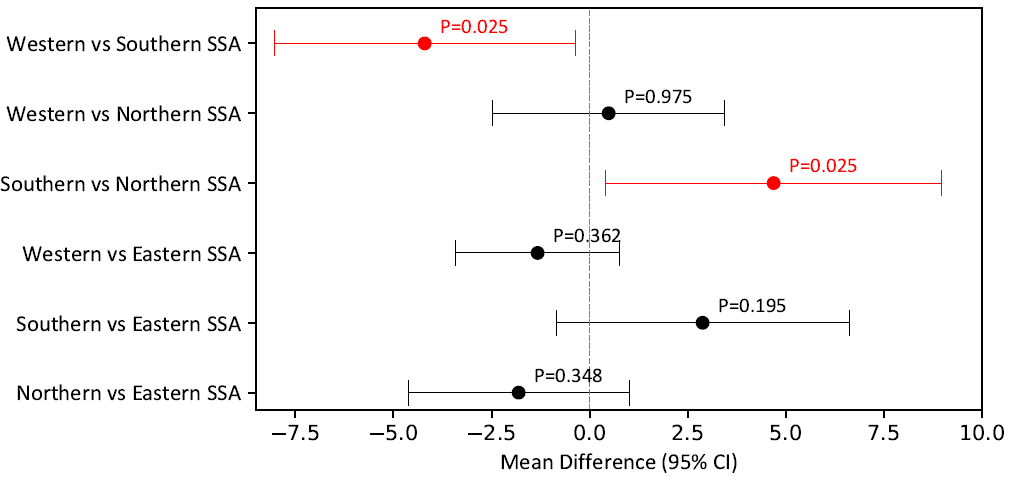


### Figure S6: Practice

##

## Supplementary Table 10: Association between distributors’ knowledge, attitude and practice

|  |  | **Knowledge** | |  |  |
| --- | --- | --- | --- | --- | --- |
|  |  | Poor | Good | Chi square | P value |
| Attitude | Negative | 171 | 193 | 97.14 | <0.0001 |
|  | Positive | 90 | 454 |  |  |
| Practice | Poor | 170 | 360 | 6.51 | 0.0107 |
|  | Good | 91 | 287 |  |  |
